# Measuring the Impact of Beamwidth on the Correlation Distance of 60 GHz Indoor and Outdoor Channels

**DOI:** 10.1109/ojvt.2021.3067673

**Published:** 2021

**Authors:** Aidan Hughes, Sung Yun Jun, Camillo Gentile, Derek Caudill, Jack Chuang, Jelena Senic, David G. Michelson

**Affiliations:** § Wireless Networks Division, National Institute of Standards and Technology, Gaithersburg, USA.; Ϯ Electrical and Computer Engineering Department, University of British Columbia, Vancouver, Canada.; ¶ RF Technology Division, National Institute of Standards and Technology, Boulder, USA.

**Keywords:** 5G, millimeter-wave, mmWave, small-scale fading, fast fading

## Abstract

Due to narrow beamwidth and channel sparseness, millimeter-wave receivers will detect much less multipath than their microwave counterparts, fundamentally changing the small-scale fading properties. By corollary, the de facto Rayleigh-Rice model, which assumes a rich multipath environment interpreted by the Clarke-Jakes omnidirectional ring of scatterers, does not provide an accurate description of this fading nor of the correlation distance that it predicts. Rather, a model interpreted by a directional ring of scatterers, recently proposed in seminal work by Va et al., theoretically demonstrated a strong dependence of correlation distance on beamwidth. To support Va’s model through actual measurement, we conducted an exhaustive measurement campaign in five different environments - three indoor and two outdoor - with our 60 GHz 3D double-directional channel sounder, compiling over 36,000 channel captures. By exploiting the super-resolution capabilities of the channel sounder, we were the first, to our knowledge, to measure correlation distance as a function of continuous beamwidth. We showed that for narrow beamwidth, correlation was maintained for much longer distances than predicted by the Rayleigh-Rice model, validating Va’s model. As the beamwidth approached omnidirectionality, with increasing number of multipath detected, the behavior indeed approached the Rayleigh-Rice model.

## I. Introduction

5G millimeter-wave (mmWave) channels experience much greater path loss than sub 6-GHz channels [1], [2]. To compensate for the loss, high-gain directional antennas are employed. Since gain is inversely proportional to beamwidth, beamwidths vary from several degrees (*pencilbeams*) to tens of degrees depending on the link budget and precise center frequency. When in motion, the transmitter (T) and receiver (R) beams will misalign quicker with narrower beamwidth, causing the received signal to decorrelate faster, translating to shorter *correlation distance* [3]. This in turn will necessitate a faster refresh rate for beamforming training [4], the time-consuming spatial channel estimation process in which the beams are realigned.

A common definition of correlation distance is the displacement over which the channel’s autocorrelation function (ACF) drops below 50% of its initial value [5]. The ACF is derived from measurements or models for small-scale (fast) fading, a phenomenon that refers to fluctuations in the received signal that occur over displacements on the order of several wavelengths due to multipath interference [6]. The de facto model for fading is Rayleigh, in which the in-phase and quadrature components of the signal are represented as i.i.d. zero-mean Gaussian random variables, sustained by the narrowband assumption for which individual paths are irresolvable (smeared). This corresponds to a rich multipath environment, interpreted geometrically by the Clarke-Jakes ring of scatterers [7], where the mobile receiver is surrounded by an infinite number of equidistant scatterers, hence have comparable strength when sensed by an omnidirectional antenna. The uniform distribution in angle-of-arrival (AoA) of the scattered paths gives rise to the classical U-shaped Doppler power spectrum [6]. The Rayleigh-Rice model is a popular variant in which a dominant scatterer, whose relative strength to other scatterers is quantified by the K-factor, is also present. The assumptions implicit to the Rayleigh-Rice model are valid for narrowband, omnidirectional systems, and thus form the cornerstone of recent channel models [8]–[10] to support the design of 4G LTE systems operating at sub 6-GHz.

The high directionality of 5G mmWave systems, on the other hand, results in the detection of only a few scatterers by the receiver. The few scatterers will in turn have a non-uniform AoA distribution [11]–[14], giving rise to an asymmetrical Doppler spectrum [15]. Furthermore, negligible diffraction at mmWave [16] makes for a sparse channel [17], leading to even fewer paths detected [11]. Consequently, the paths detected will exhibit less smearing, not just because there are few, but because the ultrawide bandwidth of mmWave enables resolution of individual paths, translating to highly correlated in-phase and quadrature components. By corollary, the assumptions implicit to Rayleigh-Rice fading do not apply to mmWave channels [18]. Surprisingly, popular models designed specifically for 5G systems [19][20] still adhere to those assumptions.

**Figure 1.**
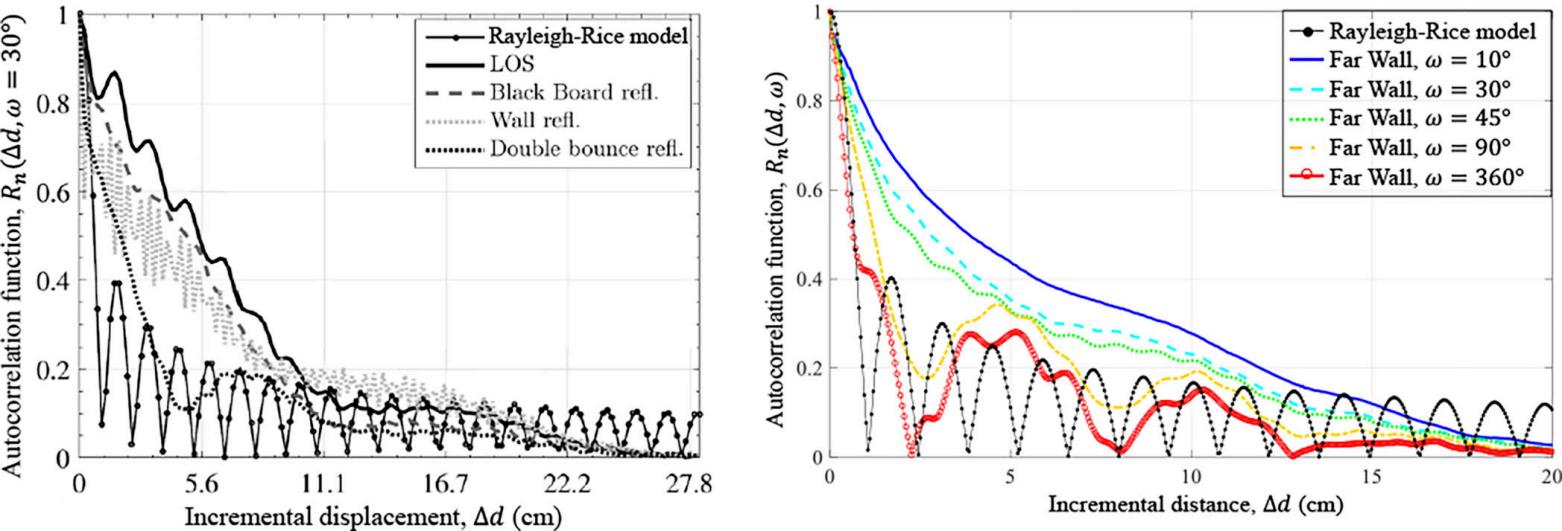
(a) Autocorrelation function (ACF) measured by Iqbal et al. [11] by mechanically steering narrowbeam (30°) horns towards select scatterers in a lecture room; the ACFs exhibited much higher correlation than the Rayleigh-Rice ACF* (b) Beamwidth-dependent ACF that we measured for the Far Wall scatterer in our Laboratory; as the beamwidth widened, more and more scatterers were admitted into the beam, and the ACF approached the Rayleigh-Rice ACF. *The formula for the Rayleigh-Rice ACF is $I_{0}\left(2\pi f_{MAX}\text{Δ}d/v\right)$, where $I_{0}$ is the zeroth-order Bessel function of the first kind. The maximum Doppler shift $f_{MAX}$ for the plot shown here corresponds to a receiver speed v=10km/h.

In [17][21][22], Pätzold theoretically investigated the correlation distance of mmWave channels, showing a significant variation across realizations with 2–10 non-isotropic scatterers. Beyond Pätzold’s theoretical work, there have been just a handful of studies on correlation distance at mmWave, and even fewer measurement-based work [2][11][23][24][25]. The 30 GHz measurements in Iqbal et al. [2], [11] employed horn antennas with $30^{\circ }$ beamwidth mechanically steered towards select scatterers in a small lecture room. The work was of notable impact because in contrast to [23]–[25], the complex form of the ACF – with no spatial averaging, neither across scenarios nor antenna elements – was computed. Fig. 1(a) shows the ACF computed for the line-of-sight (LoS) path and for the select scatterers compared to the Rayleigh-Rice ACF, demonstrating convincingly that the correlation distance of directional, mmWave channels was much longer than that predicted by Rayleigh-Rice. In addition, they demonstrated that the channels had high correlation between the in-phase and quadrature components, what is more representative of mmWave scattering conditions.

**Figure 2.**
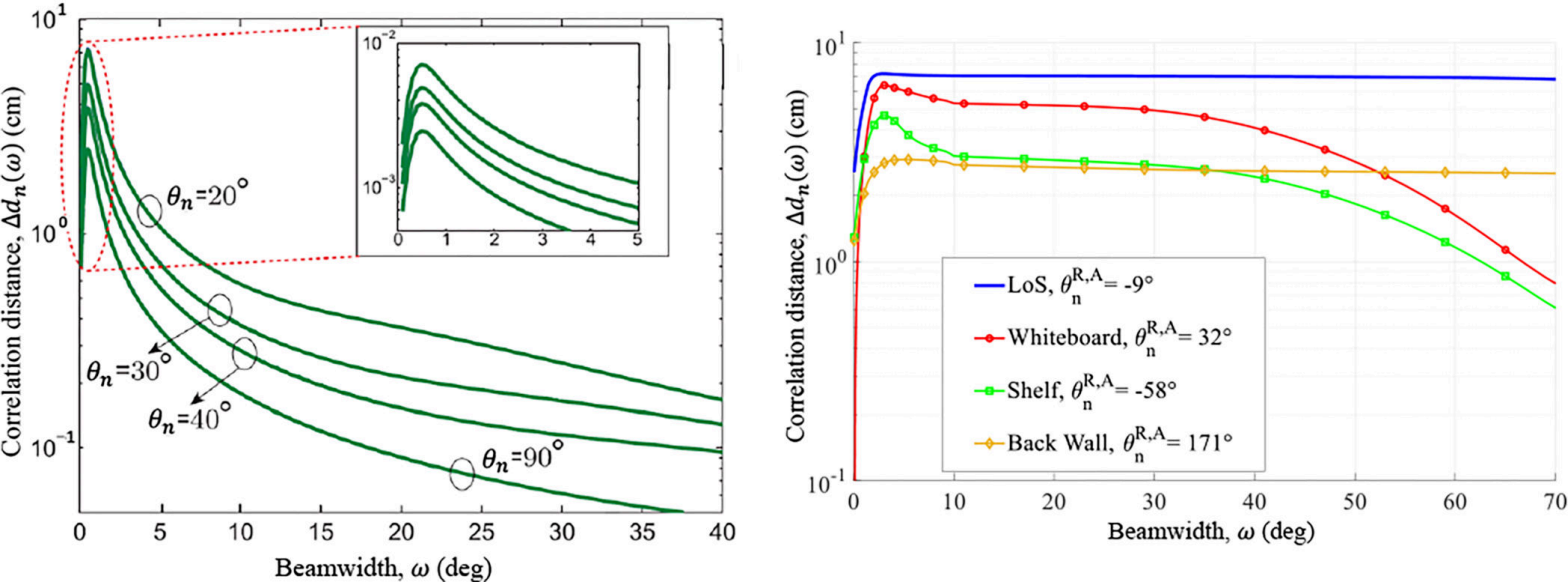
Correlation distance versus antenna beamwidth. (a) Results from Va et al.’s theoretical model [26]* for a variable-beamwidth antenna steered towards the AoA $\left(\theta_{n}\right)$ of different ambient scatterers. (b) Results from actual measurements in our Laboratory for a variable-beamwidth antenna steered towards the azimuth AoA $\left(\theta_{n}^{R,A}\right)$ of the ambient scatterers labeled in the legend, exhibiting the same behavior as Va’s model. *The abscissa was converted from correlation time in the publication to correlation distance here (for the sake of consistency) by assuming a speed of v=3.6 km/h.

Although Iqbal’s work was indeed of notable impact, the results were specific to the $30^{\circ }$ beamwidth of the horns employed for measurement, which is wider than beamwidths expected for mmWave [26]; in contrast, the 60 GHz phased-array antennas in [27] only had 3° beamwidths. For this reason, Va et al. [14], [26] investigated the impact of beamwidth, and in particular beam misalignment, on the correlation distance of vehicular mmWave channels. In their theoretical model, the receiver in the Clarke-Jakes ring of scatterers was modified to have a synthetic horn with variable beamwidth. From the model, correlation distance was computed as a function of beamwidth. See the illustrative example in Fig. 2(a): For very narrow beamwidth (ω<0.3°), the beam steered towards the AoA $\left(\theta_{n}\right)$ of different ambient scatterers became quickly misaligned as the receiver moved, causing the correlation to fall short of its peak. Widening the beam improved alignment, reaching the correlation peak around ω=0.5°, but the correlation dropped off thereafter due to more and more local scatterers being admitted into the beam. Va’s theoretical model highlighted an important characteristic of directional mobile channels, but was not supported by actual measurements.

In this paper, we bridged the gap between Iqbal’s work, which experimentally measured the ACF/correlation distance for a fixed beamwidth, and Va’s work, which modeled it theoretically for variable beamwidth. The main contributions are as follows:

1. High precision measurements with our state-of-the-art 60 GHz switched-array channel sounder, which estimated path gain, delay, and 3D double-directional angle - *i.e*. azimuth and elevation angle-of-departure (AoD) from the transmitter and AoA to the receiver - of channel paths with average errors of 1.95 dB, 0.45 ns, and 2.24°, respectively;
2. Extensive measurement campaign in five different environments - three indoor and two outdoor - comprising a total of 20 scenarios, each with 1801 small-scale acquisitions, for a total of 36,020 channel captures, enabling a comprehensive evaluation of correlation distance;
3. The first to measure correlation distance as a function of continuous beamwidth - using a single system - enabled by combining super-resolution techniques to extract paths with a synthetic horn that had variable beamwidth.

The paper is developed as follows: Section II describes our channel sounder and measurements. In Section III, the main scatterers per environment were identified and for each, the ACF was computed by steering a synthetic horn towards the scatterer. In Section IV, the correlation distance per scatterer as a function of continuous beamwidth was computed from the ACF, and representative metrics of the resultant curves were compiled over the 20 scenarios. The final section is reserved for conclusions.

## II. Channel Measurements

This section describes our channel measurements. First, our channel sounder is presented, followed by details of our measurement campaign, then by processing techniques implemented to extract channel paths and their properties from the measurements.

### A. Channel Sounder

**Figure 3.**
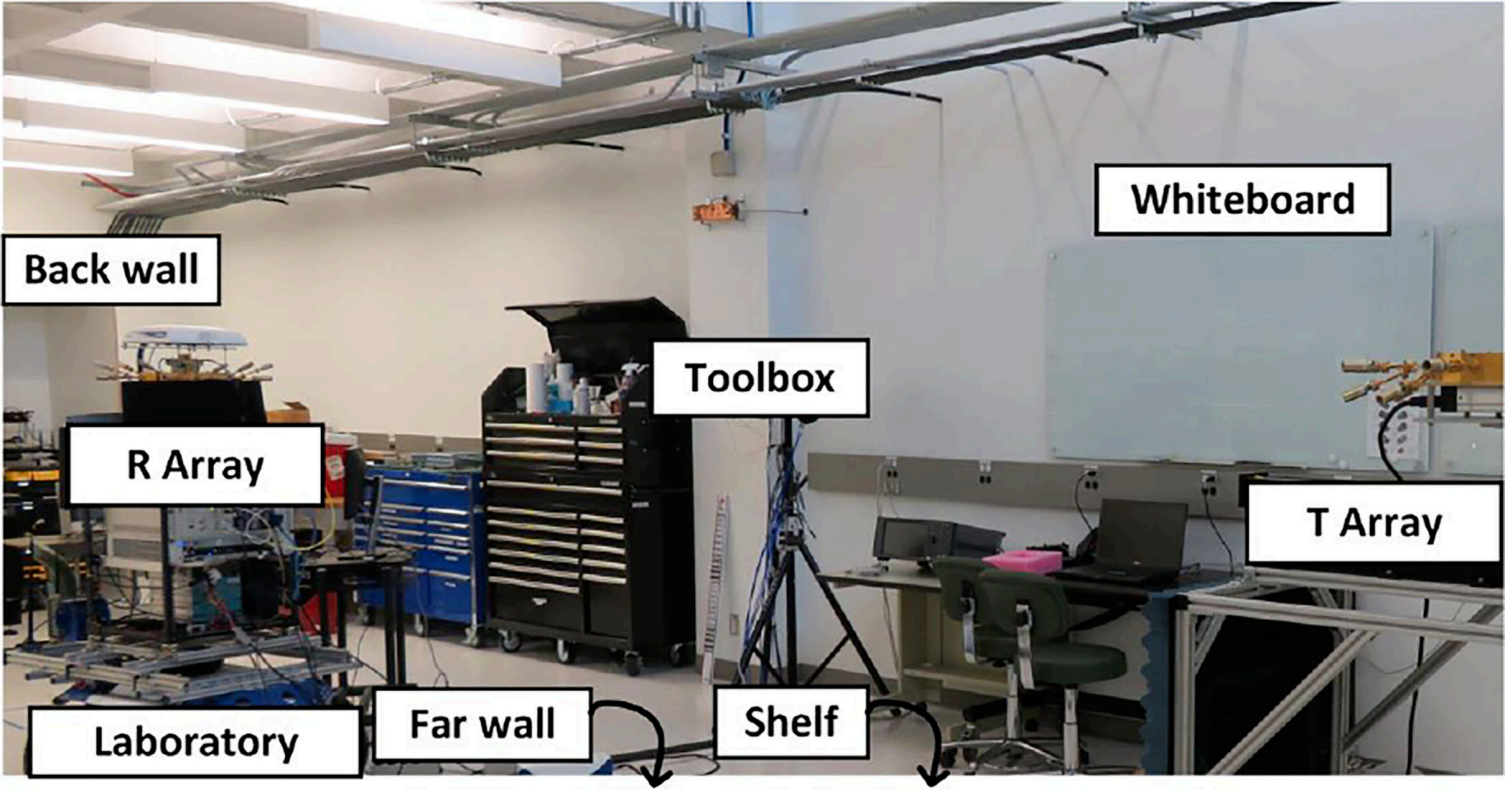
Photograph of the T array and R array of our 60 GHz 3D double-directional switched-array channel sounder collecting measurements in the Laboratory environment. Some of the main scatterers identified in the environment were labeled.

Fig. 3 displays our 60 GHz 3D double-directional switched-array channel sounder [28]. The receiver features a circular array of 16 horn antennas with 22.5° beamwidth, rendering a synthesized azimuth field-of-view (FoV) of 360° and 45° in elevation. The transmitter was almost identical except that it featured a semicircular array of only 8 horns, limiting the azimuth FoV to 180°. At the transmitter, an arbitrary waveform generator produced a repeating M-ary pseudorandom (PN) codeword, with 2047 chips of duration $T_{c}=0.5\;\text{ns}$ (or equivalent delay resolution), corresponding to bandwidth $B=1/T_{c}=2\;\text{GHz}$. The codeword was produced at IF, upconverted to 60.5 GHz, and then emitted by a horn. At the receiver, the signal received by a horn was downconverted back to IF and then sampled at 40 Gsamples/s. Finally, the sampled signal was matched filtered with the codeword to generate a complex-valued channel impulse response (CIR) as a function of delay. The codeword was electronically switched through each pair of transmitter and receiver horns in sequence, resulting in 16×8=128 CIRs, which is referred to as an *acquisition*. An optical cable between the transmitter and receiver was used for synchronous triggering and phase coherence [29]. For a transmit power of 20 dBm, the maximum measurable path loss of the system was 162.2 dB when factoring in antenna gain, processing gain, system noise, and remaining components of the link budget.

### B. Measurement Campaign

Measurements were collected in five different environments: three indoor (Laboratory, Lobby, Lecture Room) and two outdoor (Pathway, Courtyard). In each of the environments, four scenarios with two different *large-scale* T locations and two different R locations were investigated, for a total of 20 scenarios altogether. LoS conditions were maintained throughout. The T was mounted on a fixed tripod at 1.6 m height; the R was also mounted at 1.6 m on a 90 cm rail (linear positioner) whose translation was parallel to the ground. The positional tolerance of the rail was 76μm. The measurement per scenario consisted of 1801 channel acquisitions as the R was translated, equivalent to a *small-scale* displacement of $\frac{\lambda }{10}=0.05\;\text{cm}$ between each acquisition, indexed as d=0,0.05,…,90cm. Note that the minimum granularity recommended in [23] to measure small-scale fading was 4–5 samples per wavelength. It required about 30 minutes to capture all 1801 acquisitions, so static channel conditions were imposed for the whole duration (no pedestrian, vehicular motion, etc.). The indoor environments were closed off to human presence during measurement; the outdoor environments were conducted on our NIST campus, far removed from any street traffic, and before 6 am to avoid any foot traffic.

### C. Path Extraction

The 128 CIRs per acquisition were coherently combined through the SAGE super-resolution algorithm [30][31] to extract channel paths and their properties. The output from SAGE for the acquisition at displacement d was N channel paths indexed through n, together with the path properties in the six-dimensional space: complex amplitude $\alpha_{n}(d)$, delay $\tau_{n}(d)$, and 3D double-directional angle $\theta_{n}=[\theta_{n}^{T}(d)\;\theta_{n}^{R}(d)]$, where $\theta_{n}^{T}(d)=[\theta_{n}^{T,A}(d)\;\theta_{n}^{T,E}(d)]$ denoted the AoD in azimuth (A) and elevation (E) and $\theta_{n}^{R}(d)=[\theta_{n}^{R,A}(d)\;\theta_{n}^{R,E}(d)]$ denoted the AoA. The measurement error of the channel sounder was computed by comparing the extracted properties of the LoS path against its ground-truth properties, namely its 3D double-directional angle and delay given from the T-R geometry and its path gain mapped from delay through Friis transmission equation. The measurement error averaged over all displacements and scenarios was reported as 1.95 dB in path gain, 0.45 ns in delay, and 2.24° over all four angle dimensions.

Any measurement taken with a channel sounder will contain not only the response of the channel, but also the response of the sounder itself, namely the directional patterns of the antennas and the responses of the transmitter and receiver front ends. The SAGE algorithm accounted for de-embedding the antenna patterns which were characterized in an anechoic chamber, while the effects of the front ends were removed through pre-distortion filters designed from a back-to-back calibration method [28]. Hence the extracted path properties represented the “pristine” response of the channel alone and not that of the measurement system.

**Figure 4.**
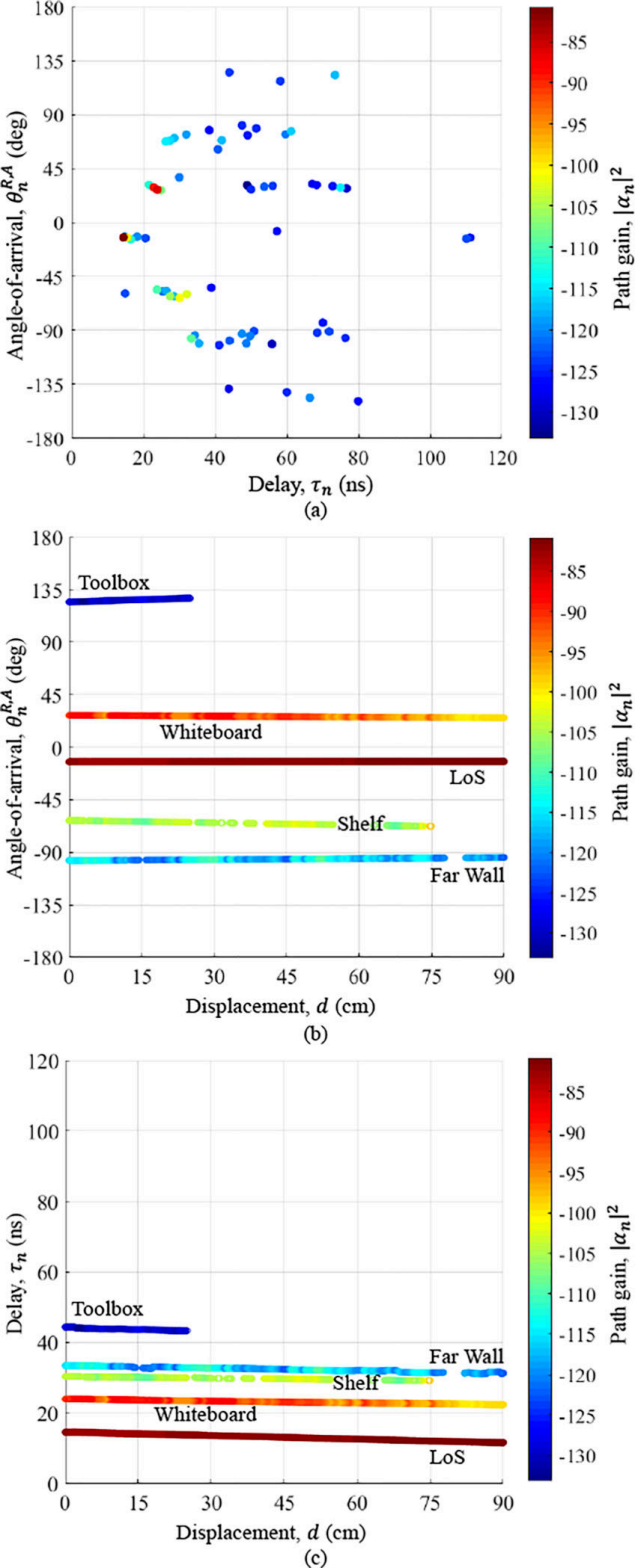
Channel paths extracted in a Laboratory scenario. *All* paths extracted at the first displacement (d=0cm) on the rail, displayed in azimuth AoA, delay, and path gain (a). *Persistent* paths only, tracked over all displacements (d=0…90cm) and labeled against environment scatterers, displayed in azimuth AoA and path gain (b) and in delay and path gain (c).

The *spatial CIR*, which incorporated the angle dimension (in addition to the delay dimension of the CIRs), offered a compact representation of the channel per scenario and could be written as

(1) $$h(d,\theta ,\tau )=\sum_{n=1}^{N}\alpha_{n}(d)\cdot p\left(\tau -\tau_{n}(d)\right)\cdot \delta \left(\theta -\theta_{n}(d)\right),$$

where p(τ) denoted the system pulse - the PN codeword after matched filtering - from a unity-gain omnidirectional antenna. It is equivalent to what a receiver (also with a unity-gain omnidirectional antenna) would detect at d, *i.e.* N copies of the transmitted pulse - each corresponding to a different path n - scaled by complex amplitude $\alpha_{n}(d)$ and arriving with delay $\tau_{n}(d)$ from angle $\theta_{n}(d)$. As an example, Fig. 4(a) displays the channel paths extracted at the first displacement (d=0cm) in a Laboratory scenario.

## III. Autocorrelation Function (ACF)

In Iqbal’s work, the CIR was acquired by mechanically steering the T horn and the R horn towards individual scatterers in a lecture room (Black Board, Wall, etc.) that were identified *a priori*. Our approach was similar, but instead of a single CIR, 128 CIRs were acquired and coherently combined to extract individual channel paths. A synthetic horn with variable beamwidth was then applied to the extracted paths to *reconstruct* a beamwidth-dependent CIR. The advantage of our approach was three-fold:

1. A beamwidth-dependent ACF (and in turn a beamwidth-specific correlation distance, described in Section IV) was computed from the beamwidth-dependent CIR, rather than an ACF specific to the beamwidth of the horns used for measurement;
2. The synthetic horn was steered towards persistent scatterers identified *a posteriori* from the acquisitions, rather than identifying presumptive scatterers *a priori*;
3. The synthetic horn was steered towards the exact angle of the scatterer identified, enabling confirmation of Va’s theoretical model for impact of misalignment on correlation distance.

Details of our approach are provided in this section.

### A. Persistent Paths

The LoS path and specular paths from ambient scatterers tended to be strong and persistent along the rail, which are desirable traits for beam steering. Diffuse paths, which clustered around specular paths in angle and delay, tended to be much weaker and could vary even over fractions of a wavelength [32]–[34], causing the total number of paths N extracted per displacement to vary along the rail despite a length of only 90 cm. Table I displays the mean and standard deviation of N along the rail per environment. The mean number was greater indoor due to more clutter (more scatterers); the Laboratory, in particular, contained many metallic instruments. The mean number was also greater indoor due to less free-space loss by virtue of smaller dimensions (shorter path lengths), enabling detection of more diffuse paths, in turn giving rise to greater standard deviation. Outdoors, variation in the number of paths was caused mostly by obstruction and scattering from foliage but diffuse scattering from buildings was also observed.

Such variation called for a robust technique to reliably identify persistent paths. To this end, paths extracted per displacement were first *tracked* along the rail using a technique based on the Assignment Problem [35], yielding *tracks* of corresponding paths across multiple consecutive displacements. While the LoS track was detected across the whole rail in all scenarios, other tracks were subject to the *birth-death* process [8]–[10], delimited by discrete birth and death displacements, denoted as $d_{n}^{BIR}$ and $d_{n}^{DTH}$ respectively - some tracks extended across just a few displacements while others extended across hundreds. Upon tracking, a persistent path was identified empirically as a path that was tracked across at least one-third of the rail (30 cm). Complete details of the tracking technique were provided in [33].

The last step was to identify the source of the persistent paths. The LoS path was easily identified as first and strongest. Specular paths were mapped against ambient scatterers by inverse raytracing their joint angle and delay to the incidence locations of salient objects visible in the maps, photographs, and 360° videos of the environments - walls, whiteboards, shelves, and pillars indoors; buildings and doorways outdoors. Fig. 4(b) and Fig. 4(c) display five persistent paths identified in the Laboratory scenario (most of them are visible in Fig. 3), in particular how the azimuth AoA and delay of the paths varied gradually along the rail, attesting to the accuracy of our system^1^. Of particular relevance to our work was the variance in the AoA - some paths varied up to a couple of degrees - capturing the impact of misalignment predicted by Va. The Toolbox, Whiteboard, Shelf, and Far Wall exhibited more variation in path properties as the reflected paths traversed their surfaces compared to the LoS path that simply propagated through air. The greater variation was due to their non-flat surfaces affecting delay and angle, and their composite materials affecting path gain. For example, the Toolbox’s recessed face with highly reflective door handles can be observed in Fig. 3. Table I also shows the average number of persistent paths per environment, which ranged between 3.8 and 5.5.

### B. Beamwidth-Dependent CIR

With the persistent paths in hand, the next step in our approach was to reconstruct the beamwidth-dependent CIR corresponding to the synthetic horn steered towards one of the paths. Consistent with Clarke-Jakes ring of scatterers, Va’s directional ring of scatterers only considered R motion (not T). The assumption inherent to both models is that all scatterers are illuminated by the T through an omnidirectional antenna. For consistency with our analysis, we made the same assumption, meaning that the synthetic horn was applied at the R only, and hence was a function of AoA only. The implication is that the AoD information extracted from our measurements was not directly exploited in the analysis. The two dimensions that AoD added $\left([\theta^{T,A}\;\theta^{T,E}]\right)$ to the six-dimensional property space of the paths was nevertheless extremely beneficial in resolving and tracking individual paths, as well as mapping specular paths against ambient scatterers.

The synthetic horn had a 3D Gaussian pattern with unity gain and half-power beamwidth (HPBW) defined in degrees by ω [36]:

(2) $$g_{n}\left(\theta^{R},\omega \right)=e^{-{\left(\frac{\theta^{R}-\theta_{n}^{R}\left(d_{n}^{BIR}\right)}{0.6\;\omega }\right)}^{2}}$$

Va’s model also applied a Gaussian beam pattern, which is accurate for the typical horn antennas [37] employed both in our measurements and Iqbal’s. The horn was steered towards $\theta_{n}^{R}\left(d_{n}^{BIR}\right)$, the AoA of persistent path n at its birth displacement, equivalent to perfect alignment. The synthetic horn was applied to the spatial CIR h(d,θ,τ) in (1) to reconstruct the beamwidth-dependent CIR as

(3) $$h_{n}(d,\omega ,\tau )=\int_{\theta^{R}}g_{n}(\theta^{R},\omega )\cdot h(d,\theta^{R},\tau )d\theta^{R}+w(d,\tau ).$$

The gain $g_{n}\left(\theta^{R},\omega \right)$ effectively attenuated some path $\overset{ˆ}{n}$ in proportion to its angular separation, $\theta_{\overset{ˆ}{n}}^{R}(d)-\theta_{n}^{R}\left(d_{n}^{BIR}\right)$, from the steering angle as the receiver moved along d, where ω controlled the roll-off. The paths were then integrated over all arrival angles $\theta_{\overset{ˆ}{n}}^{R}(d)$ and white noise w(d,τ) was added to the sample at delay τ. The sampling rate of 40 Gsamples/s matched the sampling rate of our channel sounder.

**Figure 5.**
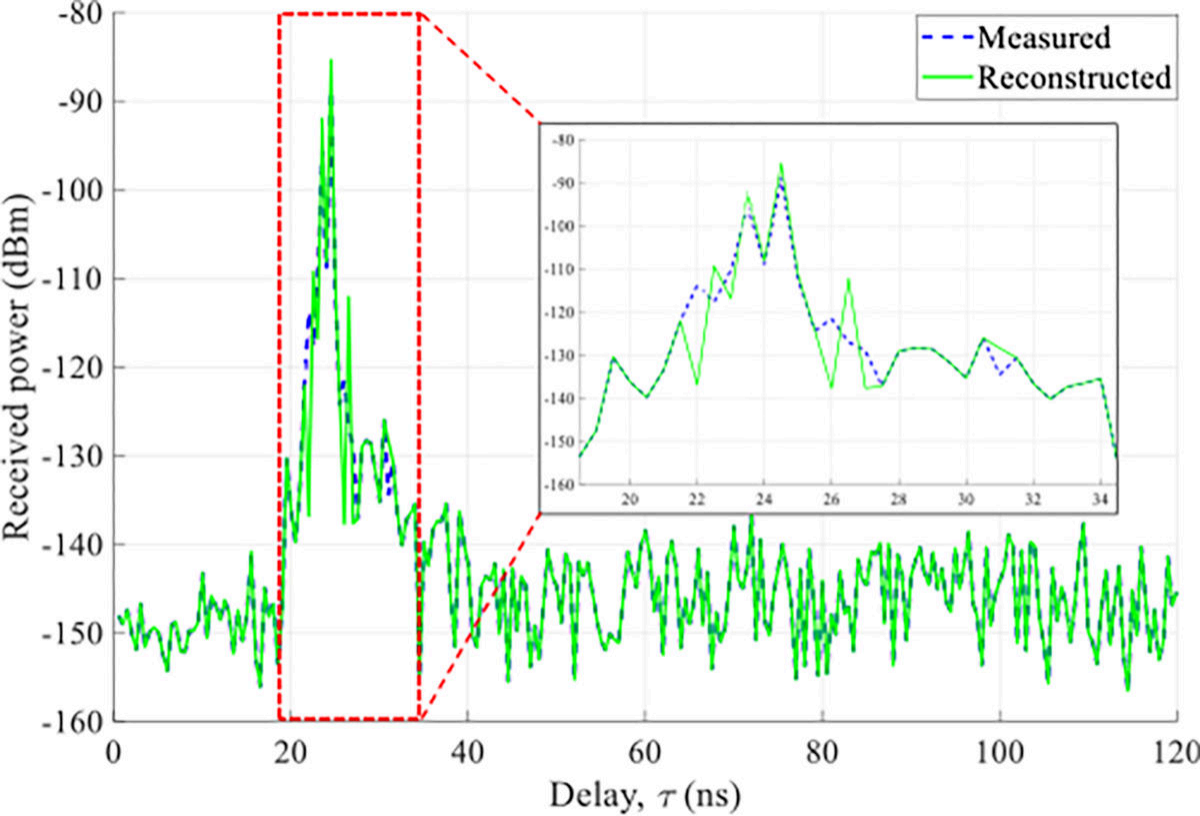
Comparison between a CIR measured with a real horn and a CIR reconstructed with a synthetic horn whose parameters (gain, beamwidth, and steering angle (orientation)) were matched to the real horn.

Fig. 5 attests to the fidelity of the reconstruction. Displayed is a CIR measured with a real horn in the Laboratory scenario together with the CIR reconstructed from the synthetic horn and the spatial CIR corresponding to the scenario. The parameters of the synthetic horn (gain, beamwidth, and steering angle (orientation)) were matched to the real horn. As can be seen, the CIR was faithfully reconstructed^2^, where the peaks correspond to channel paths.

### C. Beamwidth-Dependent ACF

Next, the complex ACF was computed from (3) as [38]:

(4) $$R_{n}\left(\text{Δ}d,\omega \right)=\frac{\int_{\tau }h_{n}\left(d_{n}^{BIR},\omega ,\tau \right)\cdot h_{n}^{*}\left(d_{n}^{BIR}+\text{Δ}d,\omega ,\tau \right)d\tau }{\int_{\tau }{\left|h_{n}\left(d_{n}^{BIR},\omega ,\tau \right)\right|}^{2}d\tau }.$$

The ACF quantified the rate at which the beamwidth-dependent CIR steered towards persistent path $n,h_{n}(d,\omega ,\tau )$, decorrelated with incremental displacement Δd along the rail. In practice, $R_{n}(\text{Δ}d,\omega )$ was computed along the path’s track, from $d=d_{n}^{BIR}(\text{Δ}d=0)$ to $d=d_{n}^{DTH}\left(\text{Δ}d=d_{n}^{DTH}-d_{n}^{BIR}\right)$. The denominator normalized the maximum value of the ACF (at Δd=0) to 1.

Fig. 1(b) displays the ACF steered towards the Far Wall scatterer in the Laboratory scenario, for various beamwidths. For ω=10°, the receiver beam was highly focused on the specular path while diffuse paths clustered locally had AoAs that were slightly offset, and were therefore admitted into the beam only marginally. As beamwidth widened, attenuation on the diffuse paths was reduced, giving rise to oscillations (widening the Doppler spread) due to multipath. The ACF can be viewed as a composite of complex-valued ACFs, each from a separate path in the beam with its own AoA and in turn its own Doppler shift, *i.e*. rate of phase rotation versus displacement [17]. As more and more paths were admitted, the oscillations intensified (the Doppler spread widened); at ω=360°, the ACF resembles the Rayleigh-Rice ACF, which in fact corresponds to the omnidirectional case of a rich scattering environment.

The oscillations could be observed thanks to the complex-valued ACFs and, in this respect, were comparable to Iqbal’s complex-valued ACFs in Fig. 1(a). Although their beamwidth was fixed at ω=30°, the various scatterers (Black Board, Wall, etc.) had different oscillations nevertheless. In their case, however, the different oscillations were not due to a synthetic horn attenuating specular and diffuse paths from the same scatterer differently, but rather the attenuation that was inherent to the various scatterers.

## IV. Correlation Distance

In this section, correlation distance as a function of beamwidth was computed for all persistent paths identified. The resultant correlation profiles were then characterized through a number of salient metrics and subsequently aggregated over the 20 scenarios for a comprehensive, statistical representation.

### A. Correlation Profiles

The original use of the term *coherence* was intended as the length of time or space over which a signal did not change appreciably and where fading was due to multipath effects only, that is, the coherent summation of paths with similar amplitudes but random phases [7]. Such fading is witnessed in rich, omnidirectional, wideband scattering environments, resulting in a *wide-sense stationarity* (WSS) channel [39], meaning that the CIR’s second-order statistics, *i.e*. its mean and variance, are constant. In turn, the Rayleigh-Rice ACF used to quantify coherence is dependent only on the incremental displacement of the receiver, not on its absolute location [6].

The *spatial stationarity* of a channel is a concept different than WSS, and refers to the spatial stationarity of persistent paths that arises from environment geometry, forming *stationarity regions* [40] or *local regions of stationarity* [9] in popular channel models for MIMO. While recent measurements have demonstrated that WSS does not hold for sparse, directional, wideband channels [41] in which persistent paths are dominant - this was also confirmed with our own measurements - the ACF has nonetheless been employed to quantify the rate at which these channels decorrelate with displacement [3][23][24][38][41][42]. Although these papers still use the term coherence distance, we feel that the more appropriate term to capture general stationarity, *i.e*. wide-sense and spatial, is *correlation distance*. Accordingly, the correlation distance of the signal, which in our application was the beamwidth-dependent CIR steered towards persistent path n, was defined as the incremental displacement where the signal’s ACF first fell below 0.5, computed from (4) as:

(5) $$\text{Δ}d_{n}(\omega )={\left.\text{Δ}d\right|}_{R_{n}(\text{Δ}d,\omega )=0.5}.$$

The upper bound on the correlation distance corresponds to the best case scenario, for which the channel only has one path - the persistent path n and no other multipath. In this case, the upper bound is determined by two factors:

1. *Bandwidth*: the wider the signal bandwidth (B), the narrower the pulse, and the faster the signal decorrelated when in motion;
2. *Misalignment*: the greater the angle $\theta_{n}^{R}=[\theta_{n}^{R,A}\;\theta_{n}^{R,E}]$ between the path and the receiver motion (the direction of receiver motion was conveniently set to $\theta^{R}\equiv \left[\begin{array}{ll} 0^{\circ } & 0^{\circ } \end{array}\right]$ in our coordinate system), the faster the decorrelation.

Given these two factors, the correlation distance is bound by (see Appendix):

(6) $$\begin{array}{ll} \text{Δ}d_{n}(\omega ) & \leq 0.5\cdot \frac{c}{B}\cdot \left|\text{cos}\theta_{n}^{R,A}\cdot \text{cos}\theta_{n}^{R,E}\right|\\ & \leq 0.5\cdot \frac{c}{B}\cdot \frac{\left|f_{n}\right|}{f^{MAX}} \end{array},$$

where c is the speed of light. Sometimes, the upper bound is preferred in terms of the path’s Dopper shift $f_{n}$ relative to the maximum Doppler shift $f^{MAX}$, where $\frac{f_{n}}{f^{MAX}}=\text{cos}\theta_{n}^{R,A}\cdot \text{cos}\theta_{n}^{R,E}$ [43]. It follows from (6) that paths more aligned with the receiver motion inherently had longer correlation distance; in particular, the upper bound $\text{Δ}d_{n}(\omega )=0.5\cdot \frac{c}{B}=7.5\;\text{cm}$ could be achieved only in the case of perfect alignment, either when the R moved directly towards the $\text{scatterer}\;(\theta_{n}^{R}=[0{}^{\circ}\;0{}^{\circ}]),f_{n}=f^{MAX}$, *i.e*. maximum Doppler shift) or directly away from it ($\theta_{n}^{R}=[180{}^{\circ}\;0{}^{\circ}],f_{n}=-f^{MAX}$, *i.e*. minimum Doppler shift).

The correlation distance was computed versus beamwidth, tracing out a *correlation profile*. Consider the profiles in Fig. 2(b) for four persistent paths identified in a Laboratory scenario. (Note that this was for a different Laboratory scenario than in Fig. 4, underscoring that different scatterers were observed for different T-R positions due to the limited FoV of the channel sounder.) Let $\text{Δ}d_{n}\left(\omega^{MAX}\right)$ denote the *maximum correlation distance* computed, falling at the *maximum correlation beamwidth* $\omega^{MAX}$. The slight misalignment of the LoS path $(\theta_{LoS}^{R}=[-9{}^{\circ}\;0{}^{\circ}])$ caused $\text{Δ}d_{n}(\omega_{LoS}^{MAX})=7.23\;\text{cm}$ to fall short of the upper bound. Although the reflection from the wall in back of the receiver (Back Wall) had the same, but opposite, misalignment as the LoS path $(\theta_{BW}^{R}=[171{}^{\circ}\;0{}^{\circ}]),\text{Δ}d_{n}(\omega_{BW}^{MAX})=2.97\;\text{cm}$ was significantly shorter due to multipath interference from diffuse scattering, whereas the LoS path was void of diffuse scattering altogether. The other two specular paths, from the Whiteboard $(\theta_{WB}^{R}=[32{}^{\circ}\;0{}^{\circ}])$ and the Shelf $(\theta_{Shelf}^{R}=[-58{}^{\circ}\;0{}^{\circ}])$, had greater misalignment than the LoS path and the Back Wall, hence their peaks were more pronounced. As beamwidth was expanded further, more and more diffuse paths were admitted, causing a further drop in correlation.

The measured correlation profiles in Fig. 2(b) exhibited behavior similar to Va’s theoretical curves in Fig. 2(a) when expanding the beamwidth from $0^{\circ }$. The behavior was characterized by an initial peak in correlation due to the improved alignment of the beam with the persistent path. The improvement was offset however by the additional scatterers admitted into the beam that disrupted the correlation. Eventually, when the beamwidth was expanded enough, the latter effect took over, dictating the drop-off.

### B. Multiple Persistent Paths

The case investigated thus far - the same case investigated by Pätzold, Iqbal, and Va - was when the beam contained a single persistent path. In this case, the correlation distance dropped monotonically after the peak due to more and more diffuse paths being admitted to the beam. The case of a single persistent path will generally apply to antennas with narrow beamwidth, but how narrow? Moreover, what happens when other persistent paths are admitted into the beam? By expanding the beamwidth to ω=360°, eventually admitting all paths in per scenario, the aim of this subsection is to answer those questions.

**Figure 6.**
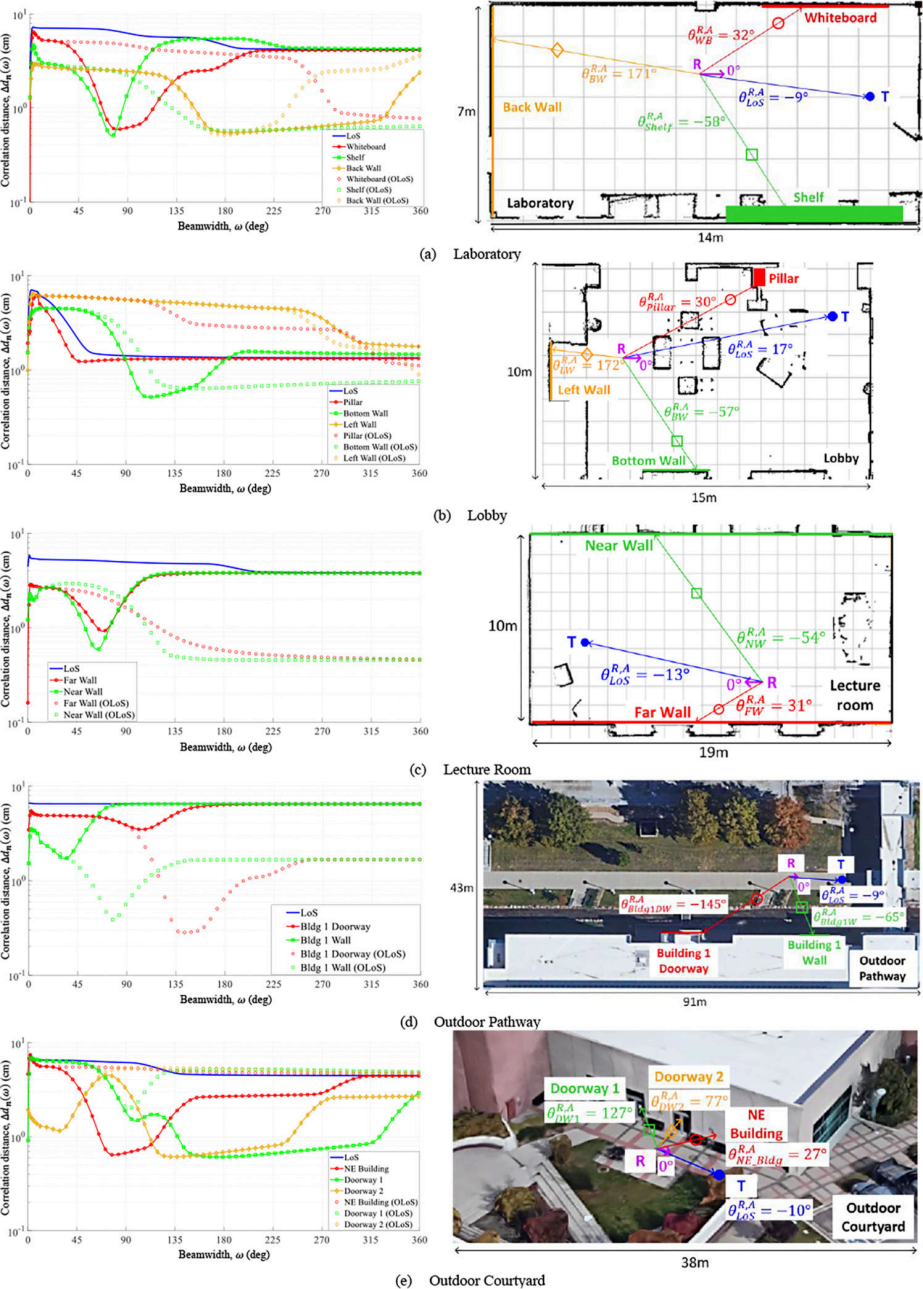
Beamwidth-dependent correlation distance for the full range of beamwidths for five scenarios, one in each environment.

Fig. 6 displays correlation profiles (symbols with lines) with the abscissa expanded to ω=360° - one illustrative scenario for each of the five environments - together with the scenario map in 2D. The direction of the receiver motion in the map is shown as a purple arrow $\left(\theta^{R,A}=0{}^{\circ}\right)$ and the azimuth AoA $\left(\theta_{n}^{R,A}\right)$ of the persistent paths are also shown, color-coded against the scatterers in the legend. In particular, reconsider the Laboratory scenario in Fig. 2(b): In the expanded view in Fig. 6(a), it can be observed that after the initial drop-off of the Shelf due to local scattering, the LoS path was admitted into the beam around ω=70°. The stronger LoS path dominated the beam, “pulling” the profile of the weaker path towards its own. Since a stronger path will generally have a longer correlation distance, this will cause the profile of the weaker path to rise back up after reaching a trough. Let $\text{Δ}d_{n}(\omega^{MIN})$ denote the *minimum correlation distance* computed, occurring at *minimum correlation beamwidth* $\omega^{MIN}$. The correlation profile of the Whiteboard was similar to the Shelf, however the Whiteboard admitted two other stronger paths along the rise back up, first the Shelf (around ω=105°) and then the Back Wall (around ω=170°), clearly indicated by positive steps in its profile.

An initial rise to maximum correlation due to misalignment, followed by a drop due to local scattering, and a subsequent rise due to other persistent paths in the beam was typical of all persistent paths per scenario, except for the strongest (LoS path). For the strongest path, rather, the initial rise was followed by a monotonic drop with negative steps along the way, each step occurring when another persistent path was encountered. What was common to all persistent paths was the *asymptotic correlation distance*, defined as the value when ω→∞ - when all beams were perfectly omnidirectional, and the N paths were attenuated identically in each beam^3^. Here, the channel behaved like a Rayleigh-Rice channel, with a single dominant path (LoS path) together with other persistent paths and their local scatterers, interpreted by the Clarke-Jakes omnidirectional ring of scatterers. The asymptotic correlation distance could be viewed as a composite of the correlation distances of the individual persistent paths weighed by their amplitudes. Accordingly, the asymptotic value typically converged towards that of the strongest path.

While the correlation profile of most paths followed the general trend described above, many deviations from this were observed in the actual scenarios. For example, in the Lobby scenario in Fig. 6(b), the LoS path’s correlation distance dropped precipitously due to the strong Pillar reflection separated from it by only 12.9°, and converged to an asymptotic value of only 1.34 cm. In the Lecture Room scenario in Fig. 6(c), the Near Wall reflection, the weakest of the three persistent paths identified, experienced two troughs due to the stronger LoS path and the Far Wall reflection. The LoS path in the outdoor Pathway scenario in Fig. 6(d) experienced no noticeable drop at all since the other persistent paths were much weaker. In the outdoor Courtyard scenario in Fig. 6(e), the four persistent paths were spaced far apart - the difference in azimuthal AoAs between successive paths were 37°, 50°, and 50° - so the asymptotic value was not reached by ω=360°. Finally, while most persistent paths identified in the scenarios were either the LoS path or specular reflections, the one from Bldg 1 Doorway in the Pathway scenario and the ones from Doorway 1 and 2 in the Courtyard scenario were actually strong diffractions from metallic door frames.

Flat segments were observed in all correlation profiles - essentially between persistent paths encountered - over which widening the beamwidth did not admit multipath significant enough to alter the correlation distance, attesting to the sparsity of the mmWave channel. In general, weaker persistent paths were more vulnerable to multipath, and hence experienced more variation across the profile.

### C. Obstructed LoS (OLoS)

We now investigate the case in which the LoS path was obstructed. This case is important since penetration loss at mmWave can be as high as 25 dB or more [1], [44], so if in mobile scenarios the LoS path is intermittently blocked by buildings, humans, vehicles, foliage, etc., it will go undetected. Although all measurements were collected in LoS conditions, OLoS conditions were created synthetically by discarding the LoS path in the spatial CIR in (1). The correlation profiles corresponding to OLoS conditions were also plotted in Fig. 6, in the same color as the persistent paths in LoS conditions, however just with symbols (no line).

The maximum correlation distance and beamwidth were essentially unchanged between LoS and OLoS conditions in all scenarios in Fig. 6 - the lined and unlined curves diverged much after the maximum correlation beamwidth - since the LoS path was never local to the other persistent paths anyway. When present, the LoS path was much stronger than the other persistent paths, pulling their correlation distances towards its own; when absent, much more variation was observed. In most scenarios, the other persistent paths actually benefited from the obstructed LoS path, maintaining correlation for a wider range of beamwidths; the only exception was for the outdoor Pathway scenario in Fig. 6(d), in which the Bldg 1 Doorway and Bldg 1 Wall had rich local scattering. When there existed a persistent path much stronger than the others in OLoS, such as the NE Building in Fig. 6(e), the strongest path mimicked the LoS path, experiencing no trough and pulling the others towards its own correlation distance. Note that in Fig. 6(e), the asymptotic correlation distance was actually longer in OLoS conditions whereas in the other four OLoS scenarios (Fig. 6(a–d)), there was no dominant persistent path so the asymptotic value settled somewhere between the asymptotic values of the individual persistent paths.

### D. Statistical Representation

**Fig. 7:**
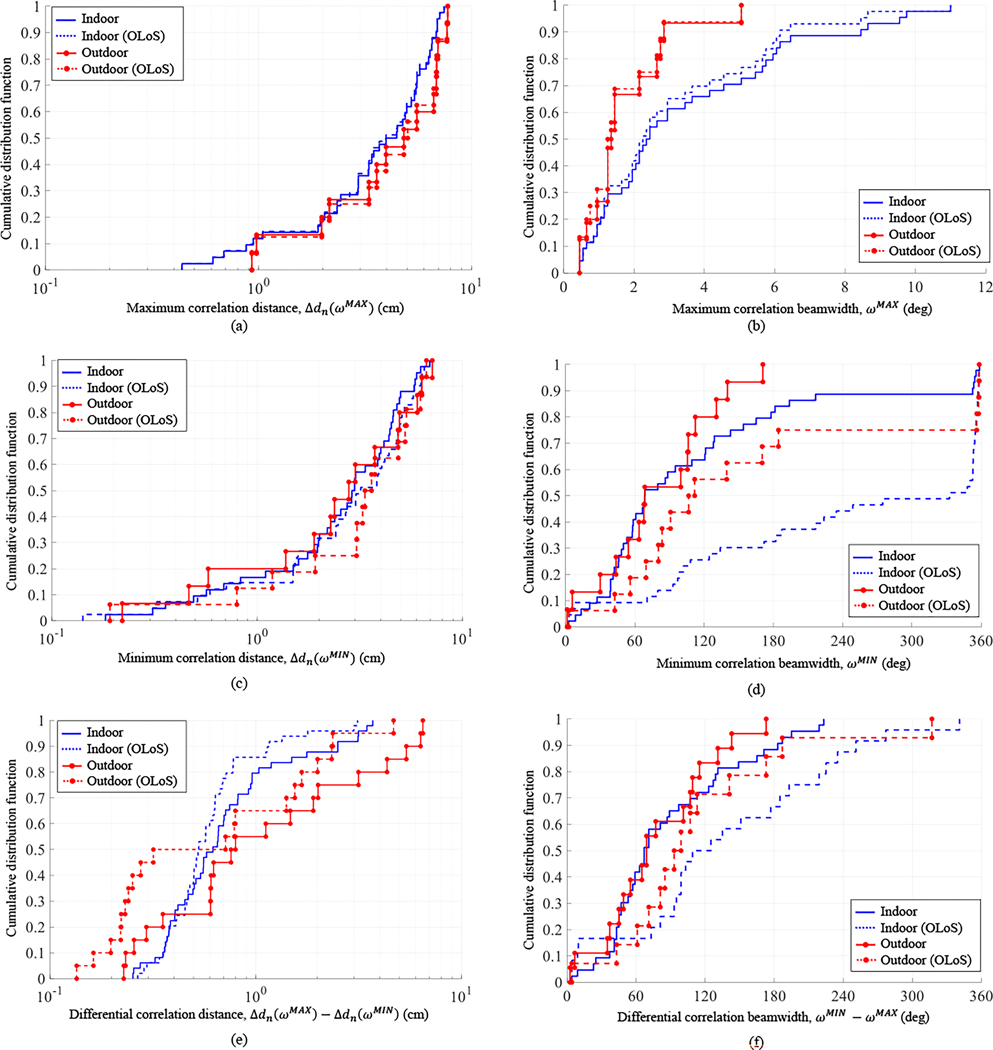
Cumulative Distribution Functions (CDFs) for salient characteristics of correlation profiles, compiled across the 20 scenarios investigated. (a) Maximum correlation distance (b) Maximum correlation beamwidth (c) Minimum correlation distance (d) Minimum correlation beamwidth (e) Differential correlation distance (f) Differential correlation beamwidth

Across the 20 scenarios considered, correlation profiles were generated for a total of 88 persistent paths in LoS conditions and a total of 68 (=88–20) persistent paths in OLoS. As demonstrated in Fig. 6, the common trend in the profiles was a rise to maximum correlation followed by a drop to minimum correlation, both occurring at relatively narrow beamwidth. Thereafter, the profiles could vary significantly from each other, but what happens for wider beamwidths is less relevant at mmWave since it is expected that antennas will have beamwidths less than 30° [26]. As such, for the purpose of comprehensive statistical representation across all the profiles computed, the individual profiles were characterized by their maximum and minimum correlation distance and maximum and minimum correlation beamwidth. These four metrics were then compiled into Cumulative Distribution Functions (CDFs) in Fig. 7. Also compiled were CDFs for the *differential correlation distance*, defined as the maximum minus the minimum correlation distance, and for the *differential correlation beamwidth*, defined as the minimum minus the maximum correlation beamwidth. The latter two metrics characterized, respectively, the correlation distance lost due to local scattering and the beamwidth over which the loss occurred. All CDFs were partitioned into indoor and outdoor scenarios, and then further partitioned into LoS and OLoS conditions.

As explained earlier, there were three factors that affected maximum correlation distance: bandwidth, misalignment, and local scattering. Because bandwidth was equal for all scenarios and because misalignment was arbitrary, the longer maximum correlation distance outdoors versus indoors in Fig. 7(a) could be justified by poorer local scattering in part, as explained earlier, due to greater path loss, so the weakest diffuse paths went undetected. Indoors, the multipath interference from the richer scattering caused the detected AoA of the persistent path to shift from its actual AoA, requiring a wider maximum correlation beamwidth than outdoors in Fig. 7(b) to capture the path reliably when in motion.

Not only were the persistent paths stronger indoors, there were more of them (see Table I); as a consequence, the destructive interference between them was more severe, translating into shorter minimum correlation distance and wider minimum correlation beamwidth in Fig. 7(c,d). This effect was better evidenced through the differential correlation distance and beamwidth in Fig. 7(e,f), which factored out the maximum correlation distance and beamwidth. While OLoS conditions did not significantly affect the maximum beamwidth, the minimum correlation beamwidth was significantly widened in absence of the strongest (LoS) path to overtake the beam quickly in the correlation profile. The minimum correlation distance was then reached later, allowing it more time to drop, hence shorter minimum correlation distance compared to LoS.

## V. Conclusions

In this paper, we combined recent seminal works by Iqbal et al. in measuring correlation distance for a fixed beamwidth and by Va et al. in theoretically modeling correlation distance versus beamwidth, both at millimeter-wave. Specifically, we were the first, to our knowledge, to measure correlation distance as a function of continuous beamwidth, using a single channel sounder rather than multiple systems with different bandwidths. It was found that correlation was maintained for much longer than what is predicted by the Rayleigh-Rice fading model, the de facto standard for sub 6-GHz channels still widely used for millimeter-wave. It was also found that correlation was maintained for a longer distance and for a wider beamwidth outdoors versus indoors due to less multipath. Another significant finding was that obstructed line-of-sight conditions were actually conducive to higher correlation since the line-of-sight path behaved as a dominant interferer to beams steered in other directions.

## Figures and Tables

**TABLE I:** AVERAGE* NUMBER OF PATHS EXTRACTED PER ENVIRONMENT

Environment		All paths (*N*)		Persistent paths
		Mean of *N* (over rail)	Std. dev. of *N* (over rail)	Number
Indoor	Laboratory	74.5	4.3	5.0
	Lobby	24.9	2.4	4.8
	Lecture Room	35.5	5.1	5.5
Outdoor	Pathway	11.1	1.5	3.8
	Courtyard	15.3	2.1	4.0

* Average over the four scenarios per environment

**Fig. 8:**
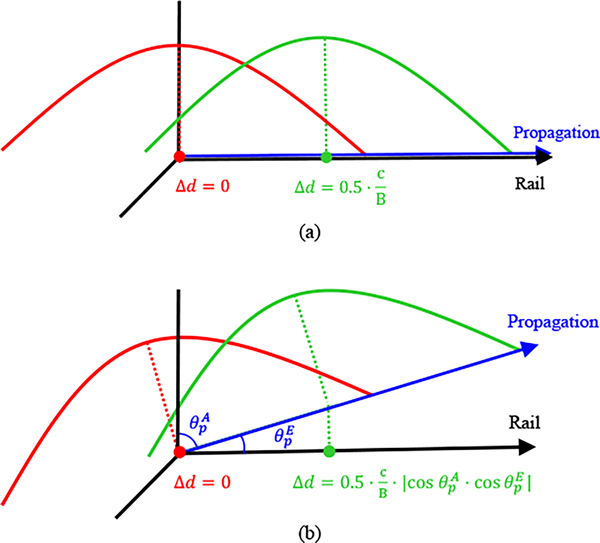
Correlation distance of the system pulse p(τ). (a) Special case where the pulse’s propagation direction is aligned with the rail. (b) General case where pulse propagates in direction $\theta_{p}=[\theta_{p}^{A}\;\theta_{p}^{E}]$ with respect to the rail.

## Footnotes

1 The inherent measurement error of the path properties per displacement was reduced by averaging the properties over the tracks through a sliding window.

2 The actual noise from the measured CIR was used to fill in samples in the reconstructed CIR that had no paths.

3 Note that since 𝜔 denotes half-power beamwidth, the pattern is not perfectly omnidirectional at 𝜔=360°.

## Appendix

Here we compute the ACF of the system pulse p(τ) (see Section II.C), assuming that the channel has only one path whose propagation is represented by the pulse. The ACF has the same format as (4):

(7)
$$R_{p}(\text{Δ}\tau )=\frac{\int_{\tau }p(\tau )\cdot p^{*}(\tau +\text{Δ}\tau )d\tau }{\int_{\tau }|p(\tau ){|}^{2}d\tau }.$$

Its computation is depicted in Fig. 8, where the pulse received at incremental displacement Δd=0(p(τ), red) is autocorrelated with the pulse received at some other Δd along the rail (p(τ+Δτ), green). For the special case in Fig. 8(a), the direction of propagation is aligned with the rail, so the incremental delay is given simply as $\text{Δ}\tau =\frac{\text{Δ}d}{c}$. By substituting in (7), the ACF could be written conveniently in terms of Δd as:

(8)
$$R_{p}^{ALIGN}(\text{Δ}d)=\frac{\int_{\tau }p(\tau )\cdot p^{*}\left(\tau +\frac{\text{Δ}d}{c}\right)d\tau }{\int_{\tau }|p(\tau ){|}^{2}d\tau }.$$

According to (5), the pulse’s correlation distance was defined as where its ACF first fell below 0.5, or

(9)
$$\text{Δ}d_{p}^{ALIGN}={\text{Δ}d|}_{R_{p}^{ALIGN}(\text{Δ}d)=0.5}.$$

The actual pre-distorted pulse (see Section II.C) was used to solve (9) numerically, resulting in:

(10)
$$\text{Δ}d_{p}^{ALIGN}\approx 0.5\cdot \frac{c}{B^{\prime }}$$

where the correlation distance depended only on the pulse width (given from the system bandwidth B).

For the general case depicted in Fig. 8(b), where the pulse propagates in direction $\theta_{p}=[\theta_{p}^{A}\;\theta_{p}^{E}]$ with respect to the rail, the incremental delay projected along the rail is $\text{Δ}\tau =\frac{\text{Δ}d}{c}.\left|\text{cos}\theta_{p}^{A}\cdot \text{cos}\theta_{p}^{E}\right|$, resulting in the generalized expression for the ACF:

(11)
$$R_{p}(\text{Δ}d)=\frac{\int_{\tau }p(\tau )\cdot p^{*}\left(\tau +\frac{\text{Δ}d}{c}\cdot \left|\text{cos}\theta_{p}^{A}\cdot \text{cos}\theta_{p}^{E}\right|\right)d\tau }{\int_{\tau }|p(\tau ){|}^{2}d\tau }.$$

The general expression for the pulse’s correlation distance followed as:

(12)
$$\text{Δ}d_{p}\approx 0.5\cdot \frac{c}{B}\cdot \left|\text{cos}\theta_{p}^{A}\cdot \text{cos}\theta_{p}^{E}\right|.$$
